# An Acoustic OFDM System with Symbol-by-Symbol Doppler Compensation for Underwater Communication

**DOI:** 10.1155/2016/7528353

**Published:** 2016-02-29

**Authors:** Tran MinhHai, Saotome Rie, Taisaku Suzuki, Tomohisa Wada

**Affiliations:** ^1^Graduate School of Engineering and Science, University of the Ryukyus, No. 1 Senbaru, Nishihara, Okinawa, Japan; ^2^Department of Information Engineering, University of the Ryukyus, No. 1 Senbaru, Nishihara, Okinawa 9030213, Japan

## Abstract

We propose an acoustic OFDM system for underwater communication, specifically for vertical link communications such as between a robot in the sea bottom and a mother ship in the surface. The main contributions are (1) estimation of time varying Doppler shift using continual pilots in conjunction with monitoring the drift of Power Delay Profile and (2) symbol-by-symbol Doppler compensation in frequency domain by an ICI matrix representing nonuniform Doppler. In addition, we compare our proposal against a resampling method. Simulation and experimental results confirm that our system outperforms the resampling method when the velocity changes roughly over OFDM symbols. Overall, experimental results taken in Shizuoka, Japan, show our system using 16QAM, and 64QAM achieved a data throughput of 7.5 Kbit/sec with a transmitter moving at maximum 2 m/s, in a complicated trajectory, over 30 m vertically.

## 1. Introduction

Underwater acoustic communication has been receiving a lot of attention recently since it facilitates new industries and applications such as deep-sea mining, ocean monitoring, and submarine communication. We use acoustic OFDM and particularly aim to a vertical link communication between a robot at the sea bottom and a mother ship in the surface. One of the greatest challenges we encountered is the severe time varying Doppler shift. First, due to low propagation speed of sound in water 1500 m/s, the frequency offset caused by Doppler is significant compared to subcarrier space. For example, it might be up to 50% of subcarrier in our system. Second, while high frequency acoustic signal provides high data speed, it is attenuated quickly over short distance. Usually, to cover a range of few kilometers, frequency carrier around 24 kHz is used. Therefore, in many cases, acoustic OFDM systems can be considered as a wideband OFDM system since bandwidth and carrier frequency are comparable. For example, we utilize a carrier frequency of 24 kHz which is three times of system bandwidth of 8 kHz. To support further distance, lower carrier frequency is used, and so the system bandwidth is getting closer to the carrier frequency. Consequently, each subcarrier experiences a different amount of Doppler shift depending on the position of the subcarrier, that is the position-dependent Doppler shift or the so-called nonuniform Doppler. Methods for mitigating the time varying Doppler shift can be classified in to two main groups.

In radio wireless communication, state-of-the-art methods for mitigating the time varying channel were proposed in [1–5]. In short, those methods are capable of dealing with a Doppler spread up to 10% of subcarrier space and consider a general channel model with many delay paths, and each path has a different Doppler. However, in our case, the Doppler shift might be up to 50% of subcarrier space, and all paths have a similar Doppler shift. Therefore, applying the state-of-the-art methods in radio wireless communication is not reasonable.

In underwater wireless communication, a class of method called resampling based method has been proposed recently in [6–13]. The resampling based method considered the time expansion/compression caused by Doppler and used resampling in the time domain to compensate Doppler. A typical resampling based method was proposed in [11] and achieved impressive experimental results. However, since this method resamples a data frame of many OFDM symbols with a single factor, it does not work well when the velocity changes roughly over OFDM symbols. In addition, this method requires storing an entire data frame of many OFDM symbols to estimate the time expansion/compression. Most importantly, the Doppler effect does not always manifest itself significantly in time expansion/compression, so high accuracy estimation and time scale are not easy. For example, a moving speed of 1 m/s causes a Doppler shift of 16 Hz equivalent to 16% of subcarrier space (*f*_0_ = 93.75 Hz), but the expansion/compression is only 1 (sample/an OFDM symbol).

Our proposal overcomes the drawbacks of the resampling based method. Our ideas are using continual pilots (CP) to track Doppler shift over each OFDM symbol and compensating Doppler symbol-by-symbol in the frequency domain rather than resampling in time domain. Though velocity changed roughly during pulling/pushing transmitters in our experiments, our method tracked and compensated Doppler shift well. However, the maximum Doppler shift can be estimated by using CP as 1/3 of subcarrier space in our case. To boost the estimation range, we propose a solution through monitoring the drift of Power Delay Profile (PDF) over OFDM symbols. The Doppler effect causes time expansion/compression; thus, when the FFT window is fixed at receivers PDF will be drifted over time. By measuring the drift amount of PDF, Doppler shift can be roughly estimated. Following the rough estimation is a fine Doppler shift estimation using continual pilots.

Then, the final estimation of Doppler shift is used in two stages to remove impacts of the Doppler shift. In the first stage, a simple phase derotation is performed before FFT. In the second stage, with an assumption that all delay paths has a similar Doppler rate, we separate impacts of Doppler shift from multipath channel and derive an ICI matrix which represents the position dependent Doppler shift. The ICI matrix is constructed using only the final estimation of Doppler shift and does not require estimation of channel transfer function yet. Thus, ICI is removed before channel estimation that is different from conventional ICI matrix in [1–5]. Overall, simulation and experiments results show that our method outperforms the typical resampling method [11].

The rest of this paper is organized as follows.  Section 2 presents signal model. The proposed system is presented in Section 3.  Section 4 presents simulation and experimental results. Finally, conclusion is given Section 5.

## 2. Signal Model

The transmitted signal of OFDM symbol *m* can be written as

(1) St=Re∑k=−NNDk,mej2πfc+kf0t+mTsb+TGIwith  0≤t≤T0,Tsb=T0+TGI.

Here, *T*_0_ and *T*_sb_ is the symbol length of an OFDM symbol excluded and included guard interval, respectively, and *T*_GI_ is the guard interval length. Totally, (2*N* + 1) subcarriers are utilized to carry information data. *f*_*c*_ and *f*_0_ are carrier frequency and subcarrier space, respectively. *D*(*k*, *m*) denotes data at subcarrier *k* of OFDM symbol *m*. The relative moving between transmitters and receivers caused a Doppler rate as

(2) $$\begin{array}{c} \Delta \left(\;t\;\right)=\frac{v\left(t\right)}{C}. \end{array}$$

Here, *v*(*t*) is the relative moving speed between a transmitter and receivers, and *C* ≈ 1500 m/s is propagation speed of sound in the water. The time varying Doppler rate Δ(*t*) is assumed constant within two successive OFDM symbols but changes over OFDM symbols. In addition, we assumed that there are *L* multipaths; each path has a gain of *r*_*i*_ and a delay of *τ*_*i*_, and all paths have a similar Doppler rate Δ. Thus, the received pass-band signal is written as follows:

(3) $$\begin{array}{c} R_{PB}\left(\;t\;\right)=\overset{L}{\underset{i=1}{\sum }}S\left(\;\left(\;1+\Delta \;\right)\left(\;t-\tau_{i}\;\right)\;\right). \end{array}$$

In the time domain, the Doppler effect manifests itself in time expansion/compression that is Δ*N*_*s*_ *T*_*s*_ over an OFDM symbol. Here, *N*_*s*_ is number of discrete samples of an OFDM symbol, and *T*_*s*_ is a sampling period. A straightforward idea is resampling the distorted signal to compensate the Doppler effect [6–13]. Different from those methods, we analyze and compensate impacts of the Doppler in frequency domain. After downconversion, the received signal at baseband can be written as

(4) RBBt=∑k=−NNHk,mDk,mej2πkf01+Δt︸non-uniformDoppler  shift·ej2πfcΔtβm︸commonphase  rotation,

(5) βm=ej2πfcΔmTsb+TGI,

(6) Hk,m=∑i=1Lrie−j2πfc+kf01+Δτiej2πkf0ΔmTsb+TGI.

As in (4), all subcarriers experience a common frequency offset as *e* ^*j*2*πf*_*c*_Δ*t*^. Each subcarrier experiences a different frequency offset of as *e* ^*j*2*πkf*_0_Δ*t*^ depending on the position of the subcarrier. This is called position-dependent frequency offset, or the so-called nonuniform Doppler shift in [12]. The position-dependent frequency offset significantly degrades performance of high modulation such as 64QAM. In our case, a moving speed of 1 m/s causes the common Doppler shift of 16 Hz which is equal to 16% of subcarrier space. In addition, the edge subcarriers corresponding to *k* = ±40 suffers a frequency offset of ±2.5 Hz which is equivalent to 2.5% of subcarrier space. The central subcarrier *k* = 0 does not experience this kind of frequency offset. Therefore, we must take position-dependent frequency offset into account. Estimation of the Doppler rate and compensation of its impacts are presented in the next section.

## 3. The Proposed System

**Figure 1:**
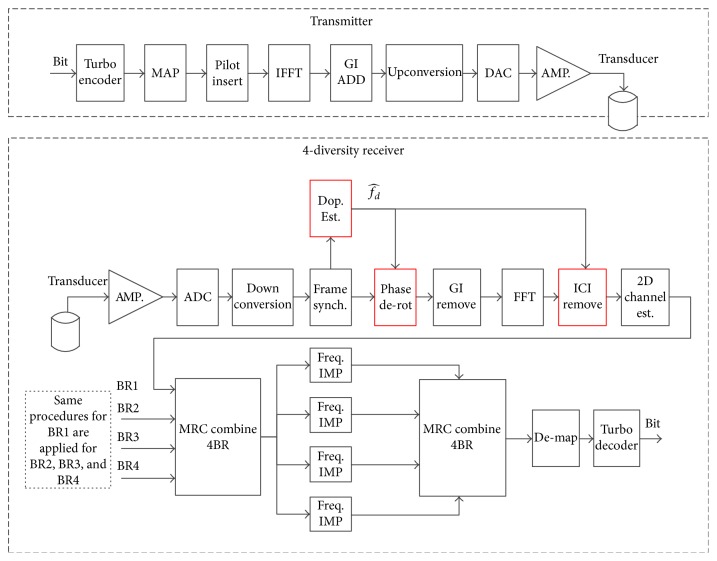
Proposed transceiver architecture.

Transducers attached to the bottom of the ship swing due to ocean wave and the robot at the sea bottom also move in a complicated trajectory. Therefore, time varying Doppler shift changes over OFDM symbols. To support such a case, the highlight of our system are time varying Doppler shift estimation, and symbol-by-symbol nonuniform Doppler compensation. In addition, impulsive noise cancellation [14–16] also is incorporated in our system. System parameters are shown in Table 1 and the overall system architecture is shown in Figure 1.

### 3.1. Estimation of Time Varying Doppler Shift

**Figure 2:**
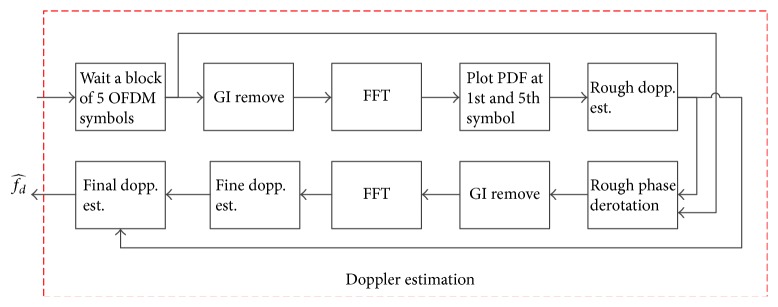
Proposed Doppler estimation.

In this section we present a time varying Doppler shift estimation by monitoring the drift of PDF in conjunction with using continual pilots. Using continual pilots can estimate Doppler shift accurately; however, as explained later the maximum Doppler shift can be estimated is limited by *f*_0_/2(1 + *T*_GI_/*T*_0_), which is *f*_0_/3 in our system. To boost the estimation range, we propose a rough Doppler estimation through monitoring the drift of PDF due to Doppler-induced expansion/compression. The signal processing of Doppler estimation is shown in Figure 2.

#### 3.1.1. Rough Estimation of Doppler Shift by Monitoring the Drift of Power Delay Profile

**Figure 3:**
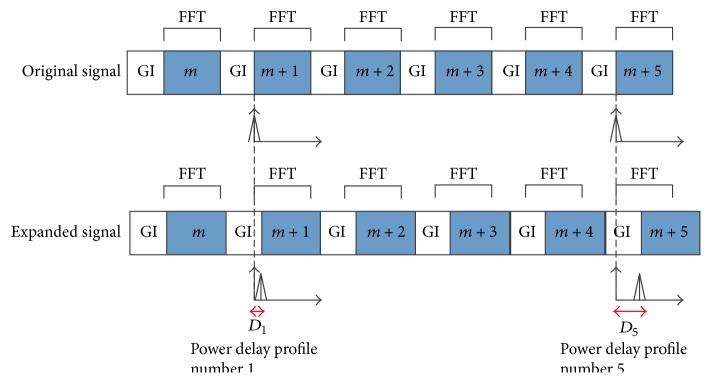
Rough Doppler estimation by monitoring the drift of PDF.

Doppler causes expansion/compression of signal wave form in the time domain. In turn, when the position of FFT window is fixed at the receiver side, we will observe the drift of PDF over OFDM symbols. This phenomenon is described in Figure 3 when the received signal is expanded. At the receiver side, PDF can be obtained from channel transfer function measured at pilot positions. PDF is obtained at symbols (*m* + 1), (*m* + 5), (*m* + 9), and so on since those symbols have more pilots then others. As shown in Figure 3, when the signal is expanded, the peak of PDF will be drifted to the right side, and the amount (*D*_5_ − *D*_1_)*T*_*s*_ indicates how much signal is expanded. Here, *D*_1_ and *D*_5_ are the drift amount of PDF measured at OFDM symbol (*m* + 1) and (*m* + 5) in scale of sample period, respectively. As mentioned above in (3) Doppler rate of Δ causes an expansion/compression of Δ*N*_*s*_ *T*_*s*_; therefore, when the expansion/compression is (*D*_5_ − *D*_1_)*T*_*s*_, the Doppler rate should be

(7) $$\begin{array}{c} \hat{\Delta_{R}}=\left(\;\frac{D_{5}-D_{4}}{4N_{s}}\;\right) \end{array}$$

and the equivalent frequency offset is

(8) $$\begin{array}{c} \hat{f_{R}}=f_{c}\frac{D_{1}-D_{5}}{4N_{S}}. \end{array}$$

#### 3.1.2. Fine Estimation of Doppler Shift by Continual Pilots

**Figure 4:**
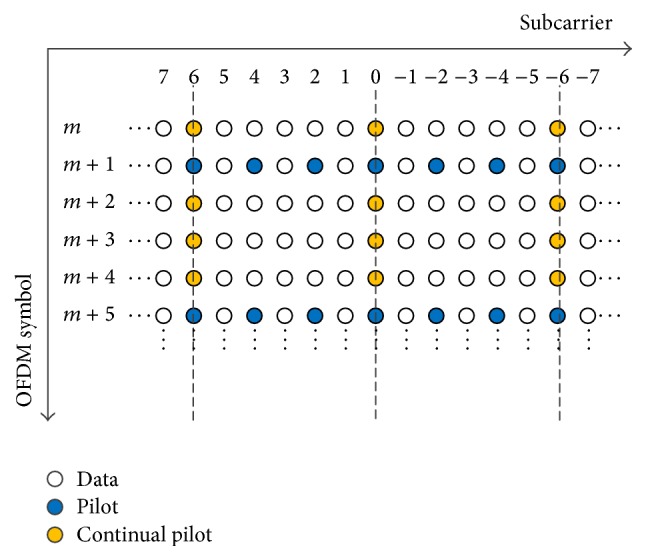
Pilot structure.

The accuracy of the rough estimation depends on the sampling speed. The smallest expansion/compression over 4 OFDM symbols can be detected as a sample period *T*_*s*_, and the corresponding smallest frequency offset can be detected as *f*_*c*_/4*N*_*s*_. Therefore, we need a fine estimation of Doppler shift following the rough estimation. After the rough estimation, Doppler is alleviated by a phase derotation using the estimated $\hat{\Delta_{R}}$. Then, the residual frequency offset in each OFDM symbol is estimated by using continual pilots. Structure of scattered and continual pilot are shown Figure 4. *C*_*p*_ is the subcarrier indexes of continual pilots in a OFDM symbol:

(9) $$\begin{array}{c} C_{p}=\left\{\;\ldots ,-12,-6,0,6,12,\ldots \;\right\}\;\forall \text{OFDM symbols}. \end{array}$$

After the phase derotation, the baseband signal in (4) is rewritten as

(10) RBB1t=∑k=−NNHk,mDk,mej2πkf01+Δt︸non-uniformDoppler  shift·ej2πfcΔ−ΔR^tγm︸commonphase  rotation,γm=ej2πfcΔ−ΔR^mTsb+TGI,fe=fcΔ−ΔR^.

After performing FFT demodulation on the received signal *R*_*BB*_^(1)^(*t*), the received signal at subcarrier *k* of symbol *m* is written as

(11) Yk,m=Hk,mDk,mγmejπkΔ+fe/f0+∑l=−Nl≠kNHl,mDl,mγmsincπl−k+lΔ+fef0ejπl−k+lΔ+fe/f0︸ICI,

(12) Yk,m≈Hk,mDk,mγmejπkΔ+fe/f0.

Similarity, the received signal at subcarrier *k* of OFDM symbol (*m* + 1) is calculated as

(13) Yk,m+1≈Hk,m+1Dk,m+1γm+1ejπkΔ+fe/f0.

To estimate the residual Doppler shift *f*_*e*_, we consider the phase difference in the channel transfer function *H*(*k*, *m*) and *H*(*k*, *m* + 1). We denote

(14) Wk,m=Yk,mDk,m,k∈Cp,Wk,m+1=Yk,m+1Dk,m+1,k∈Cp.

It is worth noting that

(15) conjHk,mHk,m+1=conjHk,mHk,m+1ej2πkf0ΔTsb,

(16) conjγmγm+1=ej2πfcΔ−ΔR^Tsb.

With *k* ∈ *S*_*p*_, *D*(*k*, *m*) and *D*(*k*, *m* + 1) are preknown continual pilots at the receiver side. From (13)–(15) the residual Doppler shift can be estimated as

(17) fe^f02π1+TGI/T0·∠⁡∑k∈CpconjWk,mWk,m+1.

Due to the angle −*π* ≤ *∠*{∑_*k*∈*C*_*p*__conj(*W*(*k*, *m*))*W*(*k*, *m* + 1)} ≤ *π*, the maximum Doppler shift can be estimated as *f*_0_/2(1 + *T*_Gi_/*T*_0_). It is noted that the continual pilots are placed symmetrically over the central subcarrier as in (9). Therefore, the element *e* ^*j*2*πkf*_0_Δ^ will be self-called in (17). The total frequency offset $\hat{f_{d}}$ is sum of rough and fine estimate values. The estimate frequency offset will be used for phase derotation, and for constructing the ICI matrix in the next section:

(18) fd^=fR^+fe^,Δ^=fd^fc.

### 3.2. Symbol-by-Symbol Doppler Compensation

#### 3.2.1. Phase Derotation

We rewrite the received after downconversion from (4) as

(19) RBBt∑k=−NNHk,mDk,mej2πkf01+Δt︸non-uniformDoppler  shift·ej2πfcΔtβm︸commonphase  rotation.

The first stage is compensating the common phase derotation using the estimation of Doppler rate $\hat{\Delta }$. Since the estimation of Doppler rate $\hat{\Delta }\approx \Delta$, after phase derotation we get

(20) $$\begin{array}{c} R_{BB}^{\left(2\right)}\left(\;t\;\right)=\overset{N}{\underset{k=-N}{\sum }}H\left(\;k,m\;\right)D\left(\;k,m\;\right)\underset{\begin{array}{c} non\text{-}uniform\\ Doppler\;shift \end{array}}{\underset{︸}{e^{j2\pi {kf}_{0}\left(1+\Delta \right)t}}}. \end{array}$$

#### 3.2.2. ICI Cancellation for Nonuniform Doppler

After performing FFT demodulation on *R*_*BB*_^(2)^(*t*), the received data at subcarrier *k* of OFDM symbol *n* is calculated as

(21) Yk,m=Hk,mDk,mejπΔk+∑l=−Nl≠kNHl,m·Dk,msincπl−k+lΔejπl−k+lΔ.

In matrix form,
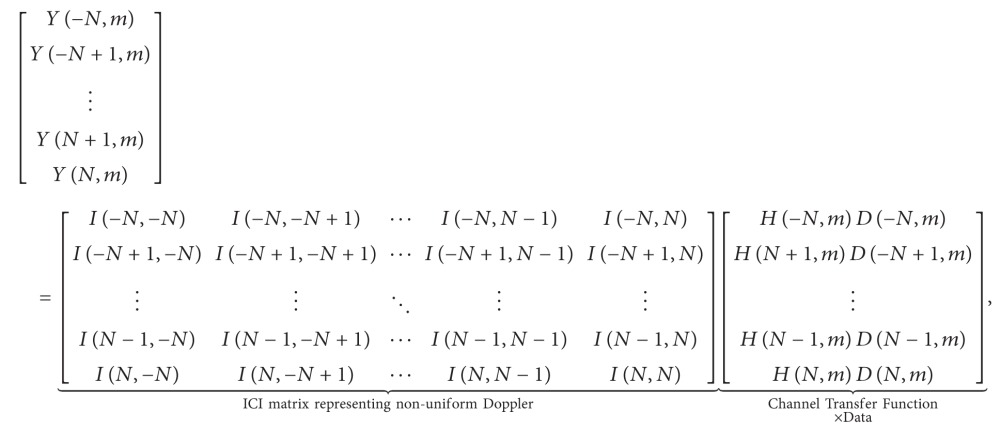
(22)

(23) Ik,l=ejπΔkif  k=lsincπl−k+lΔejπl−k+lΔif  k≠l

*I*(*k*, *l*) represents the amplitude of intercarrier interference from subcarrier *l* to subcarrier *k*. It is noted that *I*(*k*, *l*) not just depends on Doppler rate Δ, and distance (*l* − *k*) between two subcarriers, but also depends on position of subcarrier *l*. In another word, edge subcarriers suffer severe ICI compared to center subcarriers. Using the estimation of Doppler rate $\hat{\Delta }$, the ICI matrix can be determined as

(24) $$\begin{array}{c} \hat{I}\left(\;k,l\;\right)=\left\{\begin{array}{cc} e^{j\pi \hat{\Delta }k} & if\;\;k=l\\ sinc\left(\pi \left(l-k+l\hat{\Delta }\right)\right)e^{j\pi \left(l-k+l\hat{\Delta }\right)} & if\;\;k\neq l. \end{array}\right. \end{array}$$

ICI is removed before channel estimation as

(25) $$\begin{array}{c} \left[\;Y_{ICI}\;\right]=\frac{\left[Y\right]}{\left[\hat{I}\right]}. \end{array}$$

Here,

(26) $$\begin{array}{c} Y_{ICI}\left(\;k,m\;\right)=H\left(\;k,m\;\right)D\left(\;k,m\;\right). \end{array}$$

After ICI is removed from received signal, the free-ICI signal *Y*_ICI_(*k*, *m*) is fed into the estimation block and the remained channel transfer function *H*(*k*, *m*) can be easily estimated by using pilots, and 1-Taps equalizer is sufficient to recover data *D*(*k*, *m*):

(27) $$\begin{array}{c} \hat{D}\left(\;k,m\;\right)=\frac{Y_{ICI}\left(k,m\right)}{\hat{H}\left(k,m\right)}. \end{array}$$

Here, $\hat{H}\left(k,m\right)$ is estimation of channel transfer function provided by the channel estimation block.

### 3.3. Reduction of Computational Complexity

The resampling based method requires arbitrary rate resampling. Lagrange interpolation can be applied to perform arbitrary rate resampling, and coefficients of the Lagrange polynomial can be computed through finding inversion of the Vandermonde matrix. On the other hand, our rough estimation and the fine estimation have complexity of O(*n*log⁡*n*) since these procedures utilize FFT to measure the PDF at OFDM symbols *m*, *m* + 4, *m* + 8, and so on, and FFT demodulation to compute *Y*(*k*, *m*) and *Y*(*k*, *m* + 1) in (12) and (13). However, the ICI cancellation requires finding the inversion of the ICI matrix, so this procedure has complexity of *O*(*n* ^3^). To avoid matrix inversion, we can apply the Jacobi iterative method for the big ICI matrix equation (22), and the complexity is reduced to *O*(*n* ^2^). By applying the Jacobi iterative method for (22), the initial value of [*Y*_ICI_] is computed as

(28) $$\begin{array}{c} \left[\;Y_{ICI}^{\left(0\right)}\;\right]=\frac{\left[Y\right]}{diag\left(\hat{I}\right)}. \end{array}$$

Then, at the second iteration,

(29) $$\begin{array}{c} \left[\;Y_{ICI}^{\left(1\right)}\;\right]=\frac{\left[Y\right]-\left\{\left[\hat{I}\right]-diag\left(\hat{I}\right)\right\}\left[Y_{ICI}^{\left(0\right)}\right]}{diag\left(\hat{I}\right)}. \end{array}$$

[*Y*_*ICI*_^(1)^] is fed into the channel estimation and then 1-Taps equalizer to recover the data [*D*].

## 4. Simulation and Experimental Results

In this section, simulation and experimental results confirm that our proposal yields a better performance than the resampling based method. It is worth noting that all Bit Error Rate (BER) shown in the following is before Turbo decoding. After Turbo decoding, our system achieves free error in both simulation and experiments.

### 4.1. Simulation Result

#### 4.1.1. Performance of the ICI Cancellation for Nonuniform Doppler

**Figure 5:**
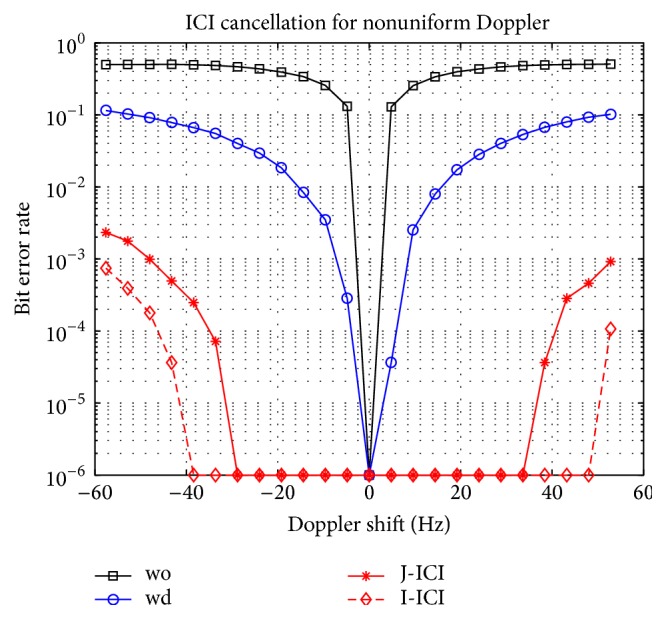
Performance of ICI cancellation for nonuniform Doppler.

The channel condition is shown in Table 2, and all paths have a similar Doppler rate Δ. In Figure 5, “*wo*” means without Doppler compensation. “*wd*” means Doppler compensating using only the diagonal of the ICI matrix, which is similar to [17]. “I-ICI” finds the inversion of the ICI matrix, and “J-ICI” utilizes Jacobi iterative method.

#### 4.1.2. Comparison between Proposed Method and Resampling Method

In this section, we compare the proposed method against a typical resampling method in [11]. In short, the resampling method resamples the whole data frame of many OFDM symbols with the same scale, then a phase derotation is employed to compensate the residual Doppler. The resampling method utilizes LFM (Linear Frequency Modulation) pre-/post-ample for Doppler rate estimation and must store an entire data frame to estimate the Doppler rate.

**Figure 6:**
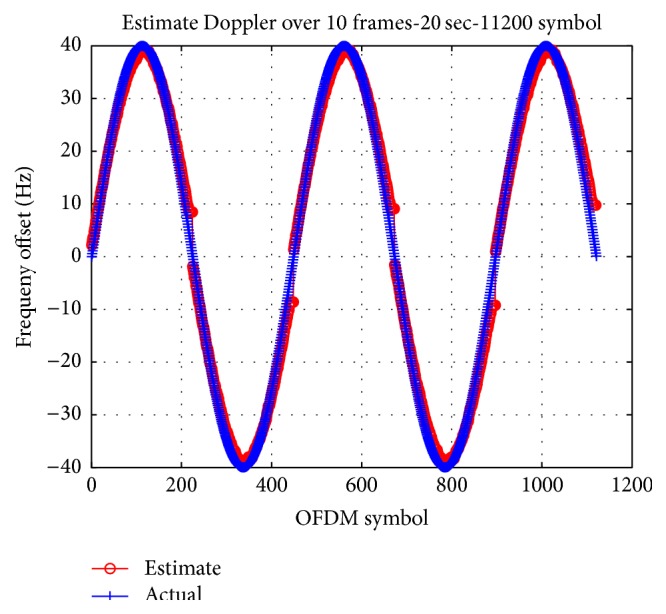
Simulation of Doppler estimation for both 16QAM and 64QAM.

Table 3 shows a channel model used in our simulation for two cases. In the first case, the maximum velocity is 2.5 m/s which causes a Doppler shift of 40 Hz and changes over time as shown in Figure 6 for both 16QAM and 64QAM. The velocity increases from zero to 2.5 m/s with an acceleration rate around 1 m/s/s which is reasonable when considering the fluctuation of transmitters due to ocean waves, or pulling/pushing transmitters in our experiments. As shown in Figure 6, though the maximum Doppler shift as 40 Hz is greater than *f*_0_/3, our tracking method using continual pilots in conjunction with monitoring PDF works well.

**Figure 7:**
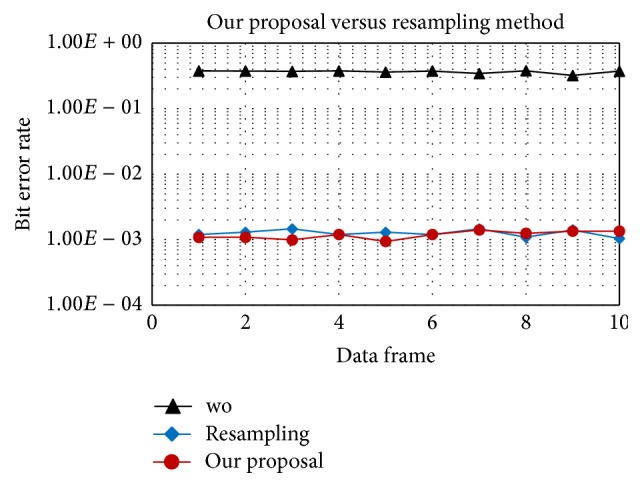
16QAM, proposed method versus the resampling based method.

**Figure 8:**
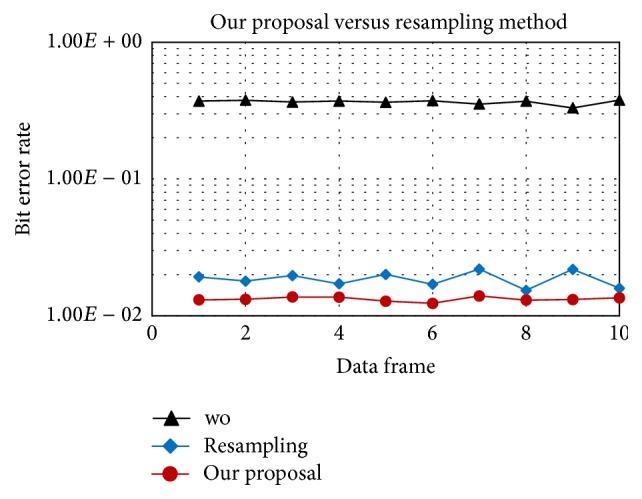
64QAM, proposed method versus the resampling based method.

In Figures 7 and 8, “*wo*” means without Doppler compensation. As mentioned before, resampling an entire data frame with a single factor does not fully compensate the time varying Doppler. After resampling, the residual position-dependent Doppler still degrades performance. In contrast, our proposal can track and compensate the Doppler shift symbol-by-symbol, provides a stable performance, and outperforms the resampling method as shown in Figure 8. In detail, with the same acceleration rate of 1 m/s/s, after resampling the residual position-dependent Doppler can be negligible for 16QAM and, however, quite significant for 64QAM. Therefore, symbol-by-symbol Doppler compensation provides gain for 64QAM as in Figure 8.

### 4.2. Experimental Result

**Figure 9:**
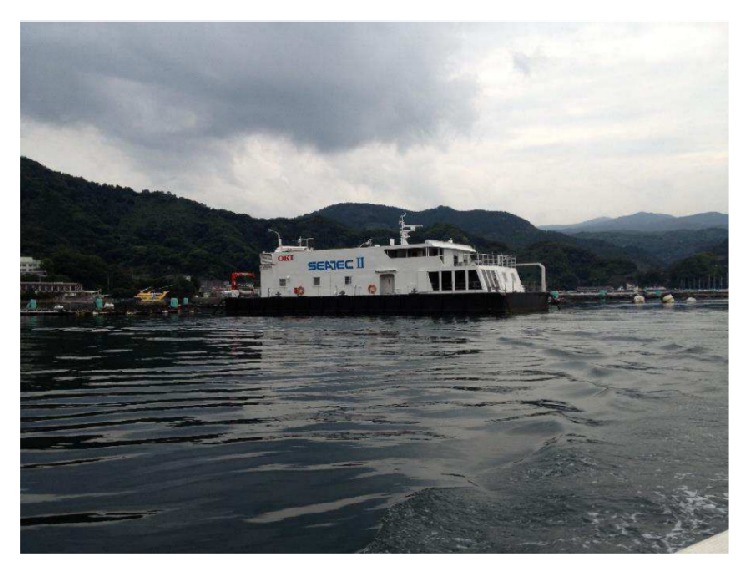
The barge.

**Figure 10:**
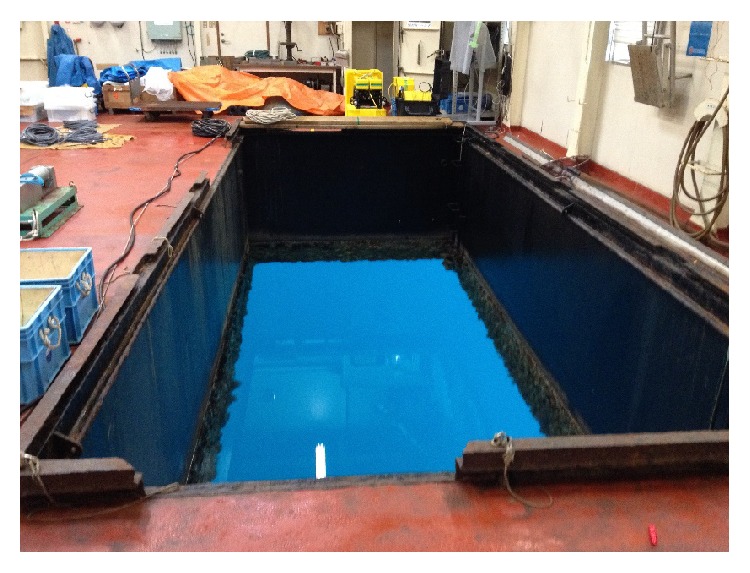
The moon pool inside the barge.

**Figure 11:**
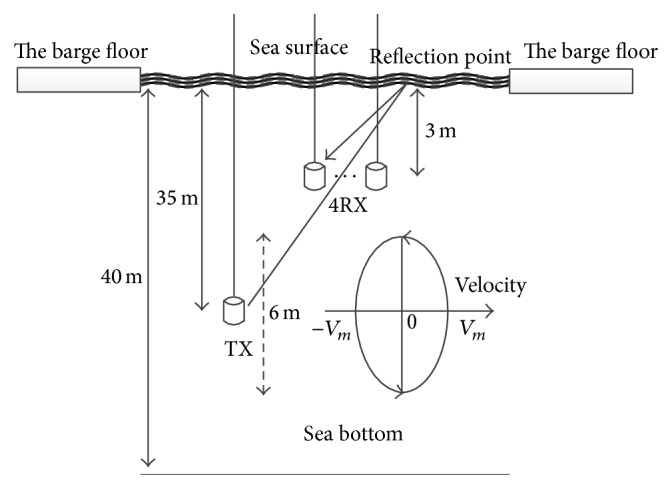
The experiment setting.

Experiments were conducted inside a barrage in January 2015, in Shizuoka, Japan. There is a moon pool inside the barge as shown in Figures 9 and 10 so that transducers can be dragged down into the water. By a controllable electric motor, a transmitter was periodicity pulled toward the surface and then pushed toward the bottom as in Figure 11. When approaching the surface or the bottom, the velocity of the transmitter decreases gradually, changes direction, and increases again. So the Doppler shift changes roughly over OFDM symbols in Figures 13 and 14. At the receiver side, four transducers are fixed at 3 m depth from the surface, and those transducers are spaced equally 10 cm apart from each other. This space is greater than the wavelength of acoustic signal in our cases, so the received signal at four transducers is uncorrelated and provides diversity gain.

**Figure 12:**
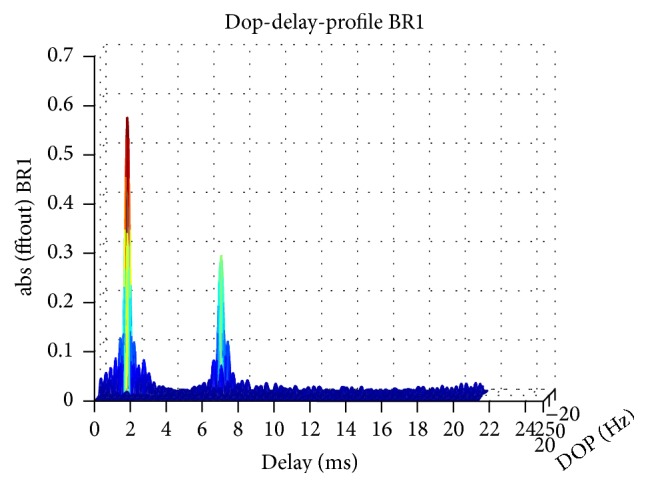
Observed power delay profile.

The estimated Delay Profile in Figure 12 is consistent with the experiment setting. In the delay profile, there is a direct path and a surface reflection path, and the distance between them is about 5 ms which is equivalent to 7.5 m distance. As the experiment setting, the receivers are at 3 m depth, and so a reflection path can appear as in Figure 11.

**Figure 13:**
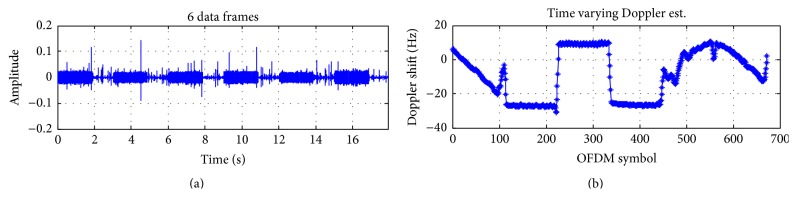
16QAM, Doppler estimation in the experiments.

**Figure 14:**
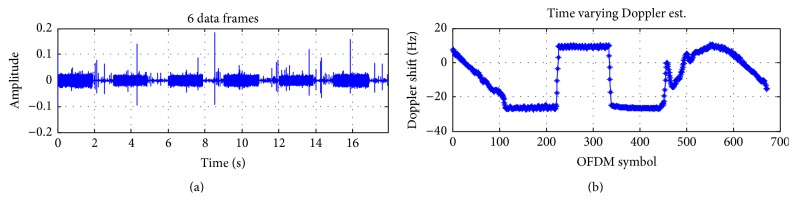
64QAM, Doppler estimation at the experiment.

**Figure 15:**
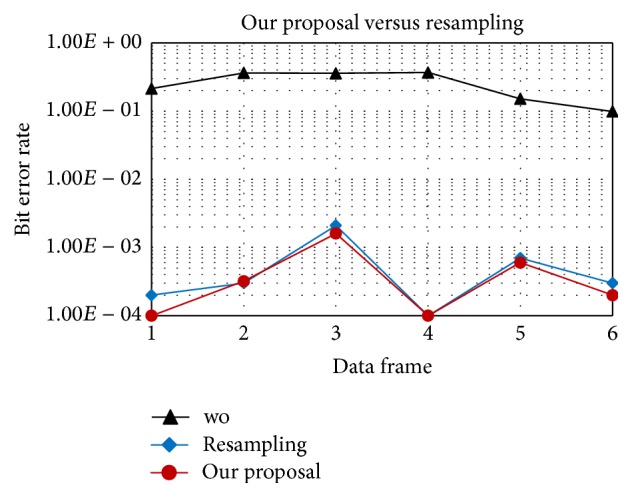
16QAM, BER of proposed method versus the resampling based method in the experiments.

**Figure 16:**
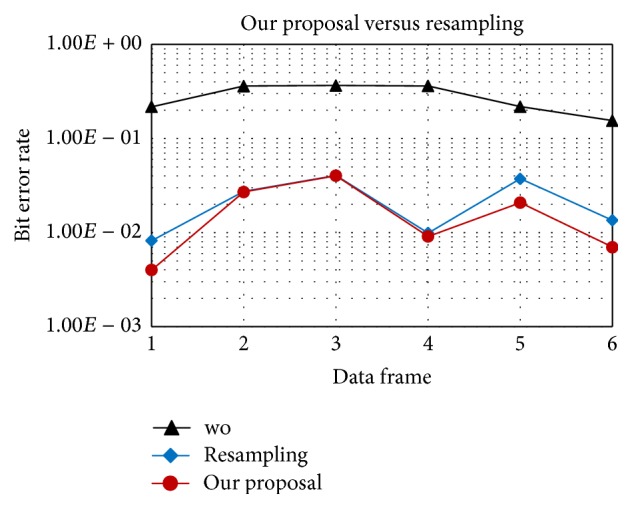
64QAM, BER of proposed method versus the resampling based method in the experiments.

Figures 13 and 14 show the estimation of the time varying Doppler shift over 6 frames, with 16QAM and 64QAM, respectively. The maximum Doppler shift estimated is about 30 Hz corresponding to the velocity of 2 m/s, which is consistent with the experiment setting. BER (Bit Error Rate) over frames of 16QAM and 64QAM is shown in Figures 15 and 16, respectively. For both 16QAM and 64QAM cases, during frames 2, 3, and 4, the Doppler shift is nearly constant over a frame, so our method and the resampling method achieved quite similar performance. With frames 1, 5, and 6, the velocity changes linearly over a data frame, and our proposal slightly outperformed the resampling method. The performance gain is very small due to the Doppler shift is still small, and the acceleration rate also is small. Similar to simulation result, the performance gain for 64QAM is greater than for 16QAM.

## 5. Conclusion

We presented a transceiver architecture for underwater wireless communication using acoustic OFDM. The highlights are estimation of time varying Doppler through monitoring the drift of PDF combined using continual pilots, and symbol-by-symbol Doppler compensation through an ICI matrix for nonuniform Doppler compensation. The simulation and experimental results demonstrate the proposal system provided a stable performance in terms of BER when Doppler changes in time. Overall, a data throughput of 7.5 Kbit/sec with free-error after Turbo decoding is achieved with 16QAM and 64QAM.

## Figures and Tables

**Table 1:** Our system parameters.

Parameters	Value	
TX-RX elements	1 TX/4 RX transducer	
Sampling frequency	96000 Hz	
TX center frequency	24000 Hz	
Band width	7593 Hz	
FFT size	1024	
OFDM symbol length *T*_0_ excluded GI	10.67 ms	
GI length *T*_GI_	0.5*T*_0_	
Sub carrier spacing	93.75 Hz	
Number of subcarrier	81	
Modulation	16QAM	64QAM
Turbo code rate	1/2	1/3
Space among transducers at RX side	10 cm	

**Table 2:** Channel for simulation (CNR = 35 dB).

Path	Delay (ms)	Power (dB)	Doppler rate Δ	Doppler shift Δ*f*_*c*_ (Hz)
1	0	0	[−2.4 · 10^−3^ 2.4 · 10^−3^]	[−57.6 57.6]
2	2	−5	[−2.4 · 10^−3^ 2.4 · 10^−3^]	[−57.6 57.6]
3	4	−7	[−2.4 · 10^−3^ 2.4 · 10^−3^]	[−57.6 57.6]

**Table 3:** Channel for simulation (CNR = 23 dB).

Path	Delay (ms)	Power (dB)	Doppler rate	Doppler shift (Hz)
1	0	0	[−1.6 · 10^−3^ 1.6 · 10^−3^]	[0 40 Hz]
2	2	−5	[−1.6 · 10^−3^ 1.6 · 10^−3^]	[0 40 Hz]
3	4	−7	[−1.6 · 10^−3^ 1.6 · 10^−3^]	[0 40 Hz]
